# Investigating Science Together: Inquiry-Based Training Promotes Scientific Conversations in Parent-Child Interactions

**DOI:** 10.3389/fpsyg.2020.01934

**Published:** 2020-08-05

**Authors:** Ian L. Chandler-Campbell, Kathryn A. Leech, Kathleen H. Corriveau

**Affiliations:** ^1^Wheelock College of Education and Applied Human Development, Boston University, Boston, MA, United States; ^2^School of Education, The University of North Carolina at Chapel Hill, Chapel Hill, NC, United States

**Keywords:** parental guidance, scientific learning, informal learning, museum learning, science education, pedagogical approaches

## Abstract

This study examined the effects of two pedagogical training approaches on parent-child dyads’ discussion of scientific content in an informal museum setting. Forty-seven children (mean age = 5.43) and their parents were randomly assigned to training conditions where an experimenter modeled one of two different pedagogical approaches when interacting with the child and a science-based activity: (1) a scientific inquiry-based process or (2) a scientific statement-sharing method. Both approaches provided the same information about scientific mechanisms but differed in the process through which that content was delivered. Immediately following the training, parents were invited to model the same approach with their child with a novel science-based activity. Results indicated significant differences in the process through which parents prompted discussion of the targeted information content: when talking about causal scientific concepts, parents in the scientific inquiry condition were significantly more likely to pose questions to their child than parents in the scientific statements condition. Moreover, children in the scientific inquiry condition were marginally more responsive to parental causal talk and provided significantly more scientific content in response. These findings provide initial evidence that training parents to guide their children using scientific inquiry-based approaches in informal learning settings can encourage children to participate in more joint scientific conversations.

## Introduction

Parents, as some of children’s first learning partners, play a vital role in scaffolding children’s learning about scientific concepts (e.g., Crowley et al., 2001; Vlach and Noll, 2016; Legare et al., 2017). Through informal interaction, parents expose children to scientific content through toys and activities (Jacobs and Bleeker, 2004). Moreover, parents play an active role in fostering children’s engagement in science by modeling interest through the questions they pose to children as well as providing explanations to their children’s questions (Crowley and Callanan, 1998; Dotterer et al., 2009; Wang and Degol, 2013; Willard et al., 2019). Such explanatory talk can be especially important, as it supplies children with relevant information and can provide insight into underlying causal mechanisms that children would be unlikely to acquire through first-hand exploration (Crowley and Callanan, 1998; Shtulman and Checa, 2012; Haden et al., 2014; Vlach and Noll, 2016). Indeed, explanatory conversations between parents and their children in informal learning settings can be beneficial for scientific learning outcomes in both the short-term (Haden, 2010; Leichtman et al., 2017) and long-term (Tenenbaum et al., 2005). In the current study, we explored the impact of a brief training session on parent-child conversation in informal science learning for 4- to 6-years-old children. We target two dimensions of effective explanatory talk: the scientific *content* and the process by which it is *delivered* to the child. Below, we expand upon the importance of parent-child conversations about science before turning to our rationale for the current study.

### The Role of Parental Explanations in Early Science Learning

Although many parents understand the importance of communicating with their children about science, they vary significantly in their tendency to provide accurate, developmentally-appropriate explanations (e.g., Shtulman and Checa, 2012). Observational research in museum contexts has found that parents often provide brief, incomplete explanations when attempting to communicate scientific information (Crowley et al., 2001). There may be many reasons that parents sometimes provide incomplete explanations, ranging from parents attempting to translate complicated science concepts into developmentally-appropriate explanations or simply being judicious with their time in response to the large number of child-initiated questions (Chouinard et al., 2007; Kurkul and Corriveau, 2018; Yu et al., 2019). Another likely reason for providing incomplete explanations is that parents feel they do not have the knowledge needed to provide scientifically-accurate explanations of scientific phenomena (Crowley et al., 2001; Shtulman and Checa, 2012). Consistent with this hypothesis, evidence from multiple research studies suggests many adults, including parents, lack an accurate understanding of the content associated with many scientific domains (Jipson and Callanan, 2003; Rigney and Callanan, 2011; Shtulman and Checa, 2012; Vlach and Noll, 2016). In some cases, such incomplete or inadequate knowledge may be associated with scientific misconceptions that can then be transferred to children. For example, Shtulman and Checa (2012) found that parents often provided explanations that propagated misconceptions about the causal mechanisms of evolutionary processes, such as the inaccurate idea that animals that share similar physical features are likely to share evolutionary ancestry (see also Kelemen and Rosset, 2009; Coley and Tanner, 2012, 2015).

Parents’ possession of – or access to – accurate scientific knowledge is not a panacea. Even when adults are knowledgeable about an underlying scientific mechanism, they are often unsure how to generate a developmentally-appropriate explanation (Vlach and Noll, 2016). In the current study, we focus not only on the *content* young children hear but also on the *delivery* of such content. Our approach is based on a growing literature from both psychology and education highlighting the important role of developing “scientific habits of mind” in early science learning settings (National Research Council [NRC], 2012). For example, the *Framework for K–12 Science Education* highlights the importance of children learning about the iterative process of science and engaging in scientific activities and thinking, such as the practices of “inquiry and investigation, collection and analysis of evidence, logical reasoning, and communication and application of information” (National Research Council [NRC], 2012, p. 250). Note that focusing on the role of parent-child communication does not negate the importance of learning about and participating in the process of scientific experimentation. As noted by Dimension 1 of the *Next Generation Science Standards*, children “cannot comprehend scientific practices, nor fully appreciate the nature of scientific knowledge itself, without directly experiencing those practices for themselves” (NGSS, 2013, p. 5). Instead, our approach is drawn from social constructivist models of learning where the dyadic language serves to scaffold and support children’s science exploration (e.g., Vygotsky, 1978).

Our focus on the *delivery* of scientific content is consistent with research indicating that children are not only sensitive to the content of information when making inferences about from whom to learn but are also sensitive to the manner in which that content is delivered (Corriveau and Kurkul, 2014; Mercier et al., 2014; Kurkul and Corriveau, 2018; Mills et al., 2019). Specifically, we explore delivery of scientific content via two different pedagogical approaches they could use when participating in a scientific activity with their child. The first, a *scientific inquiry*–based approach, seeks to leverage children’s intuitive drive for seeking explanations through question-asking (Callanan and Oakes, 1992; Kelemen et al., 2014; Frazier et al., 2016; Weisman and Markman, 2017) by guiding them through a process of asking questions, experimenting, and explaining results. Scientific inquiry approaches have been extensively explored and promoted in formal educational contexts (e.g., National Research Council [NRC], 1996, 2001, 2007; Minner et al., 2010; Furtak et al., 2012) and hold promise in informal learning contexts as well (Gutwill and Allen, 2010; Vandermaas-Peeler et al., 2016, 2017, 2018). In this study, we compare this *scientific inquiry–*based approach to a more didactic approach focusing specifically on providing *scientific explanations* to the child without first prompting such explanations through question-asking. Before turning to the current study, we review prior research regarding modification of parents’ delivery of scientific content in interactions with their children as well as preliminary studies suggesting benefits in the use of scientific inquiry approaches in informal learning contexts.

### Interventions That Modify Parental Delivery of Scientific Language

In the previous section, we highlighted the reasons why parental “business-as-usual” approaches to interacting with their child focus on parents’ delivery of explanations that may or may not be scientifically accurate with less of a focus on the scientific process (e.g., Crowley et al., 2001; Shtulman and Checa, 2012). In recent years, studies of informal science learning have begun to explore other ways parents can deliver scientific content in interactions with their children in museum settings (Fender and Crowley, 2007; Benjamin et al., 2010; Gutwill and Allen, 2010; Haden et al., 2014; Willard et al., 2019). The majority of this research employs “conversation cards” to modify the ways in which parents deliver information to their child. Such conversation cards include printed instructions and prompts and are used as an explicit reminder of how to best discuss information with their child (e.g., Fender and Crowley, 2007; Jant et al., 2014). For example, some interventions include encouraging asking questions (e.g., Benjamin et al., 2010; Haden et al., 2014; Willard et al., 2019), devising or providing explanations (e.g., Fender and Crowley, 2007; Willard et al., 2019), promoting exploration (e.g., Willard et al., 2019), or engaging in multi-step learning processes (e.g., Gutwill and Allen, 2010).

Two recent studies explicitly encouraged parents to increase the number of elaborative *Wh-* questions (what, when, where, why, how) during an engineering-focused museum exhibit (Benjamin et al., 2010; Haden et al., 2014). Both studies found that parents in the question-prompting conditions were more likely to use elaborative *Wh-* questions relative to other conditions. However, *Wh-* question prompting appeared to have mixed impacts on children’s own talk. Benjamin et al. (2010) found that children in the questions-prompting instructions conditions were more likely to respond to their parents, to engage in elaborative conversations about engineering concepts, and to correctly recall information about the exhibit. In contrast, Haden et al. (2014) found no such condition differences, suggesting that manipulating parents’ delivery in the form of elaborative *Wh-* questions may not always be effective in changing children’s own scientific discourse.

A recent study by Willard et al. (2019) focused on employing more minimal conversation-card-based interventions to modify parent-child dyad interactions with a gears activity. Here, the focus was on contrasting (1) exploration of scientific stimuli (“Explore” condition) with (2) explanation of scientific observations (“Explain” condition). Parental questions predicted children’s discussion of gears in the Explain condition but not in the Explore condition. To explain this finding, the researchers concluded that, compared to parents in the Explore condition, parents in the Explain condition were likely asking particular types of questions that prompted children to discuss the scientific content at hand. However, as their coding scheme was designed to capture the frequency of questions rather than types of questions (e.g., *Wh-* questions vs. yes/no questions), potential differences in the delivery process across conditions were not explored.

In contrast to using conversation cards to modify specific elements of parental talk, other research has focused on the inquiry process as a whole (Gutwill and Allen, 2010; Vandermaas-Peeler et al., 2016, 2017, 2018). Scientific inquiry (sometimes also referred to as “inquiry science”) is most commonly defined as constructivist learning processes wherein children learn from active engagement with scientific activities that focus on observation and experimentation to answer “scientifically-oriented questions” (National Research Council [NRC], 1996, 2001, 2007, 2012; Minner et al., 2010; Furtak et al., 2012; NGSS, 2013). Meta-analyses have indicated that inquiry-based processes have multiple positive effects on children’s learning in formal educational contexts, such as increasing engagement in the learning process and drawing conclusions from observations (see Minner et al., 2010; Furtak et al., 2012, for meta-analyses of the efficacy of inquiry-based science teaching).

In informal science learning contexts such as museums, short scientific inquiry-based interventions for family groups have proven effective (Gutwill and Allen, 2010; Vandermaas-Peeler et al., 2016). For example, Vandermaas-Peeler et al. (2016) invited families to interact with museum stations designed to elicit talk about math-centric concepts. As compared to a business-as-usual control group, the families in the group who received informational signs with inquiry-focused suggestions were more likely to produce explaining and reasoning-oriented language. Moreover, children in the inquiry group provided more correct responses to their parents’ guidance prompts than the control group, suggesting that children’s learning benefitted from participating in inquiry-based informal learning interactions.

Gutwill and Allen (2010) found similarly promising results from a more involved approach to training parents in scientific inquiry processes. Prior to entering the museum exhibit, families participated in “inquiry games” designed to elicit scientific inquiry behaviors. As compared to families in several control conditions, families who participated in the scientific inquiry-based condition, “Juicy Questions,” increased the amount of explanations and interpretations when interacting with the exhibits. However, because all analyses were conducted at the level of the family and not separated by parent or child, these findings make it challenging to determine whether the child or the adult was benefitting from the intervention.

Taken together, the results from the studies reviewed above indicate promising but mixed findings for the effectiveness of interventions focusing on scientific delivery (and not just content) in supporting informal science outcomes. Whereas some research (Benjamin et al., 2010; Vandermaas-Peeler et al., 2016; some findings from Willard et al., 2019) indicates benefits to child outcomes, other research (Haden et al., 2014; other conditions from Willard et al., 2019) finds little effects of such delivery. One reason for these mixed results might be associated with limitations of conversation cards as an intervention vehicle. Although conversation cards can prompt specific types of conversational behaviors (e.g., asking more *Wh*- questions), it is challenging to convey effective delivery approaches on a card. Therefore, research on complex interaction approaches such as scientific inquiry may need more involved intervention procedures. To this end, in the current study we chose to use a brief modeled interaction for intervention training, which has been used in previous studies focusing on modifying demonstrated scientific content (Benjamin et al., 2010; Gutwill and Allen, 2010; Haden et al., 2014; Marcus et al., 2017) but has been studied less as a vehicle for modeling delivery processes (Gutwill and Allen, 2010). We explore the impact of this intervention on parental talk and children’s talk separately to confirm the effectiveness of such modeling on both dyadic partners. In addition to focusing on the impact of the intervention on the process through which information is delivered, we also explore impacts on the content of the conversation, as well as potential interactions between delivery and content.

We focused on training parental delivery of scientific *causal* content in an informal museum setting because children’s ability to understand underlying mechanisms associated with scientific phenomena is greatly influenced by their ability to explain causal relations (e.g., Gopnik et al., 2004; Legare and Lombrozo, 2014; Walker et al., 2014). Moreover, by the age of three, children begin asking a substantial number of questions focusing on causal inferences (Isaacs, 1930; Callanan and Oakes, 1992; Chouinard et al., 2007; Frazier et al., 2009, 2016; Corriveau and Kurkul, 2014), indicating that children themselves may recognize that causal content is useful for learning about the world around them. In line with this, recently published research has demonstrated that 3- and 4-years-old selectively seek out books that contain “highly causal” as opposed to books that contain “minimally causal” information (Shavlik et al., 2020). Thus, parent talk focusing on highlighting and explaining causal relations between scientific phenomena provides access to information that may be especially engaging to children and support their learning of opaque scientific content.

In the current study, we chose to specifically study children ages 4–6 years of age, as this is an early age range in which children have begun to appreciate the value of causal information (e.g., Chouinard et al., 2007) but also frequently learn in informal learning environments in interactions with their parents (e.g., Willard et al., 2019). Parent-child dyads were randomly assigned to one of two pedagogical training methods. The first, the *scientific inquiry* process approach, invited parents to deliver scientific content via inquiry processes, inspired primarily by the work of Gutwill and Allen’s (2010) juicy questions methodology. The second, the *scientific statements* approach, was based on delivering interesting scientific statements in a more didactic manner during a dyadic interaction. This second approach was chosen as didactic approaches are regarded in educational sciences as a more traditional approach to facilitating learning interactions with children and are still being investigated as potential approaches to informal science learning (e.g., Gutwill and Allen, 2010; Willard et al., 2019). By comparing the relative impacts of these approaches on parent-child scientific conversations, we aimed to add to established best practices in assisting parents when they engage in scientific activities with their children. More specifically, this study examined whether training parents in scientific inquiry approaches provides benefits for parent-child conversations beyond the more traditional business-as-usual scientific statements approach.

We asked two research questions. First, we examined the potential impacts of the scientific inquiry and scientific statements interventions on parent-level and child-level scientific talk. Second, we explored the relation between parental talk and children’s subsequent talk about the scientific concepts to assess whether this relation differs according to parents’ assigned intervention. We predicted that parents who participated in the scientific inquiry approach would be more likely overall to ask questions than parents who observed scientific statement approaches (and conversely, parents who observed the scientific statements approach would use more statements in general). We also predicted that children whose parents observed the scientific inquiry approach would be more likely to verbally respond to their parents’ talk and that when they responded, the responses would be more likely to contain scientific content.

## Materials and Methods

### Participants

Fifty-two parents (21 female) and their 4- to 6-years-old children (20 female, *M*_*age*_ = 5.43, range = 4.00–6.91) were recruited and tested as dyads in a science museum in the Northeast United States. All participants provided written consent according to standard protocols approved by the institutional review board of the corresponding author’s university. An additional five dyads were omitted from the sample; two dyads were omitted because of interference from the children’s siblings, two from video file loss, and one from experimenter error. The remaining samples of dyads were randomly assigned to one of two between-subjects conditions, *scientific inquiry* (*n* = 25) or *scientific statements* (*n* = 22). On average, parents had a high level of education, consistent with demographics of the average museum visitor (*M_education_* = 17.36 years; Soren, 2009; Dawson, 2014). No significant condition differences in education levels were found. Overall, 41 out of 47 parents reported their occupations, of whom 14 (34.15%) had a STEM-related career. No significant differences were found in the percentage of parents who were employed in STEM-related careers between the conditions (*Scientific inquiry*: 38.1%; *Scientific statements*: 30.0%). Average child continuous age (in years) did not significantly differ between conditions, *t*(45) = 0.48, *p* = 0.64.

### Testing Procedure

**FIGURE 1:**
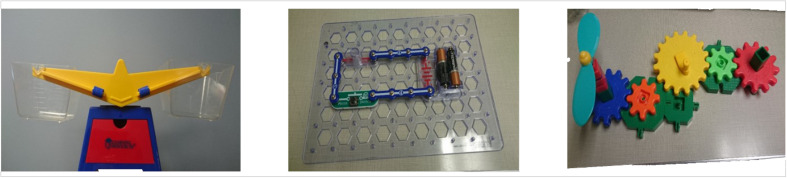
Study phases with their respective tasks.

Dyads participated in three phases (baseline, training, post-training). To introduce the tasks, the experimenter said that they were interested in “looking at how children learn through interactions with their caregivers” and asked the dyads to play together as they would at home. Interactions were videotaped for further analysis. Dyads played with three separate activities presented in a fixed order (baseline: a balance scale; training: a circuit board [Snap Circuits^©^ by Elenco]; post-training a mechanical gears layout, see Figure 1). The balance scale toy had clear plastic buckets and 74 bear-shaped colored weights. The circuit board (Snap Circuits^©^ by Elenco) consisted of three circuit pieces, a switch, a battery unit, and a lightbulb component arranged in a rectangular circuit on a circuit board. Two additional circuit pieces were on the board but not connected to the circuit. The mechanical gears were based off of Legare and Lombrozo (2014) and consisted of five differently-colored gears of varying sizes (three large and two small) attached to six green hexagonal bases, as well as a crank handle. Recordings of speech samples were transcribed offline. Below, we briefly describe the three tasks and the training conditions before turning to the transcription and coding procedure.

#### Baseline

To explore variability in dyadic interaction prior to the intervention, dyads were instructed to “play together, just like you would at home!” with the balance toy. There was no time limit; dyads interacted with the toy as long as they wished before moving on to the next two phases. The average time to complete this phase was 3.84 min (*SD* = 1.70 min, Range = 1.23–8.85 min). Marginally-significant differences were found between the *scientific inquiry* condition (*M* = 3.41 min) and *scientific statements* condition (*M* = 4.34 min) in average interaction time, *t*(45) = −1.93, *p* = 0.06.

#### Training

Next, the experimenter introduced the dyads to a previously constructed snap circuit board (see Figure 1). Dyads watched as the experimenter modeled one of two different pedagogical training conditions using interaction scripts to ensure that all parents received the same training information. As with the baseline, the training phase did not have a time limit; average completion time was 5.44 min (*SD* = 2.87 min, Range = 2.40–17.70 min). Half of the dyads were randomly assigned to the *scientific inquiry* condition. The experimenter introduced the dyad to the snap circuit and its components and explained that they would help with running a scientific investigation. The experimenter described that there were three steps to a “science investigation”: question, experiment, and explain. Then, the experimenter explained that in a science investigation, it was important to choose a question that “we don’t know the answer to” but could answer with the materials available in the moment. The experimenter invited the child to choose between one of two causal-related questions to guide the inquiry (i.e., “What happens when we take a piece out of the circuit?” or “What happens when we add a piece to the circuit?”). Next, the experimenter prompted the child to make a hypothesis about what would happen and was invited to answer the question through an *experiment* and encouraged to “try it a few times, in different ways” to understand the results. After the child completed their experiment, they were invited to *explain* the answer to the question using the evidence that they had discovered with the experiment.

The remaining half of dyads were presented with the *scientific statements* condition. The experimenter provided the child with information about the different parts of an electrical circuit, taking time to explain specific concepts and mechanisms. For example, the experimenter explained that the word “circuit” was a “fancy word for a loop” and that electricity travels from the battery, around the circuit, and makes the light turn on. The experimenter also demonstrated how pressing the switch connects the circuit, making the light turn on, but releasing the switch disconnects the circuit and makes the light turn off. The child was then informed that it was his or her turn to play and that he or she could use the extra circuit pieces on the board when doing so. Throughout this process, the experimenter explained the results of the child’s actions by referencing the previous scientific statements, linking the child’s actions and obtained results back to those statements. For example, if a child disconnected a piece from the circuit and then attempted to turn on the light by pressing the switch, the experimenter would explain how all parts of the circuit “loop” had to be connected in order for the light to turn on.

During the administration of both conditions, parents were not actively encouraged or discouraged to participate in the interaction. Regardless of parental participation, the experimenter ensured that all procedures in both conditions were completed so that the parent could observe how to implement the proposed pedagogical approach, whether it was the *scientific inquiry* or *scientific statements* approach.

#### Post-training

Finally, the experimenter invited the dyad to practice what they had just learned in the training session by playing with the gears activity. The experimenter handed the parent an information sheet for reference, which varied by condition. Both conditions prompted discussion of the same causal content (e.g., the effects of speed and direction on gear movement), but varied according to the delivery of that content. In the *scientific inquiry* condition, parents were prompted to run a science investigation with their child using the “question, experiment, and explain” method, and were provided with four possible questions the dyad might use to explore. In contrast, in the *scientific statements* condition, parents were prompted to share “scientific information” with their children, “just like I did with the circuit,” and were provided with a sheet containing scientific explanations about the gears. Parents were explicitly instructed that they were free to use as many or as few questions or statements as they liked when they interacted with their child. As with the previous two phases, there was no time limit; average completion time was 5.04 min (*SD* = 2.79 min, Range = 1.78–17.90 min). Engagement, measured as amount of time spent on the post-training gears task, did not significantly vary by training condition; dyads in the *scientific inquiry* (*M* = 4.65 min) and the *scientific statements* (*M* = 5.49 min) spent about the same amount of time on the task, *t*(45) = −1.04, *p* = 0.31. Moreover, there were no differences in the amount of child talk. Children in *scientific inquiry* produced 19.76 utterances, and children in *scientific statements* produced 20.73 utterances, on average, *t*(45) = −0.17, *p* = 0.87.

### Transcription and Coding

Interactions were transcribed at the level of the utterance for all 47 transcripts. Each transcript was verified by a second research assistant to ensure accuracy. We then removed all utterances that were directly sourced from the text of the supplemental information sheets, yielding a corpus of 3,532 utterances (2,311 parent utterances, 1,221 child utterances). All utterances were examined within the context of the entire transcript, allowing coders to read as much of the interaction as needed to ensure accuracy across the coding categories. All utterances were then coded for their *delivery* and *content*, described in more detail below.

#### Delivery

The delivery of each parent and child utterance was coded into one of two mutually exclusive categories: *question* (e.g., “How does this work?” P#1, line 21) or *statement* (e.g., “Put this on this one over here.” P#18, line 45).

#### Content

Utterances were initially coded for content, or type of information conveyed by the utterance. Content coding was initially developed based on the causal and fact-based categories used in previous studies (e.g., Chouinard et al., 2007; Frazier et al., 2009; Kurkul and Corriveau, 2018). Overall, eight types of content were coded: fact-based, causal, procedural, confirmation/negation, reinforcement, and irrelevant/unintelligible.

##### Fact-based

Fact-based utterances included content that discussed scientific facts or observations relevant to the activity. For example, a parent might observe that a gear is spinning fast (“That was super fast.” P#13, line 359) or ask how many bears are in one of the balance toy’s buckets (“So how many bears was that?” P#20, line 148). These scientific utterances included information from readily observable evidence or from the speaker’s prior knowledge of the topic. Note that fact-based statements simply describe an observation about a current state of phenomena and do not provide information or insight into *how* or *why* phenomena or events occur.

##### Causal

In contrast to fact-based utterances, causal utterances involve discussion of the relation between two or more scientific facts or observations and potentially seek to answer *how* or *why* those phenomena or events occur. These scientific utterances include the relation between cause and effect, a proposed mechanism for change, or a hypothesis or prediction based on proposed actions. For example, a parent might ask a child about why a gear is not spinning with another gear (“Why doesn’t it spin with the yellow one?,” P#46, line 78) or provide an explanation of why a smaller gear completes more rotations than a big gear when the two are connected (“and if they want to run as fast as you are they have to take a lot more steps’ cause they’re a lot smaller.” P#5, line 133).

##### Procedural

Utterances were coded as procedural when they provided on-task content regarding a stated goal, steps on what to do next, or other directives that did not contain informational content. These included directives from a parent (“Put the red one there.” P#15, line 196) or discussions of intended actions (“Should we try a different one?” P#45, line 54).

##### Confirmation/negation

These utterances were on-task but low-effort responses that provided little content beyond previous utterances. Examples include “Yes,” “No,” or “That’s right.”

##### Reinforcement

Reinforcement utterances provided motivational feedback or prompting with the goal of continuing the activity or conversation. These include everything from non-confirmatory responses (“okay” as narration of action), positive feedback (“High five, dude.” P#13, line 281) to permission granting (“Go ahead.”) and conversational fillers (“mm.” or “um.”).

##### Irrelevant/unintelligible

Any utterance that was off-task or was uninterpretable on the video recording was coded as either irrelevant or unintelligible.

Proportions of each of these categories by interlocutor (parent, child) and condition are displayed in Table 1. Because our main goal was to examine the exchange of substantive content – that is, content that provides relevant information about actions in the scientific activity, observations about the activity, or discussion of scientific phenomena relevant to the activity – the remainder of the analyses focus specifically on *causal, fact-based*, and *procedural* talk.

**TABLE 1:** Mean percentages of overall talk by content category in the post-training phase by speaker (parent, child) and condition (scientific inquiry, scientific statements).

	**Parents**			**Children**		
	**Total**	**Scientific inquiry**	**Scientific statements**	**Total**	**Scientific inquiry**	**Scientific statements**
Causal %	12.72	14.08	11.18	9.71	11.54	7.62
Fact-based %	21.31	19.15	23.78	24.69	26.42	22.72
Procedural %	29.00	30.71	27.06	18.64	17.43	20.03
Confirmation/Negation %	3.53	3.18	3.94	14.33	11.32	17.75
Reinforcement %	22.46	23.16	21.66	3.30	4.84	1.55
Irrelevant/Unintelligible %	10.88	9.60	12.33	29.33	28.45	30.33

#### Reliability

Two research assistants, blind to the training condition and the hypotheses of the study, independently coded the transcripts. Inter-rater reliability was established using 15% of the transcripts. Overall agreement was 84% for content codes (average K = 0.76) and 98% for delivery codes (average K = 0.97). Discrepancies were resolved through discussion.

## Results

Based on standard child language data-analytic techniques, we chose to pool data from dyads’ talk in the post-training phase, making the utterance, instead of the dyad, the level of the analysis. This approach is consistent with multiple previous studies (e.g., Bartsch et al., 2003; Frazier et al., 2009, 2016; Kurkul and Corriveau, 2018), and is consistent with the standards proposed by Bakeman and Gottman (1997). These standards allow for utterances to be treated as independent, assuming that coding decisions are made separately for individual utterances and the coding categories are mutually exclusive. Both of these conditions were met in the current coding scheme. Moreover, to ensure that the results were not driven by a few parent-child dyads within a condition, we followed a multi-step analytic process. First, we present mixed-effects models at the level of the utterance to explore each research question. Next, as suggested by Bakeman and Gottman (1997) we confirm that these results hold at the level of the dyad by inspecting the number of participants in each condition that reflect the pattern demonstrated by the mixed-effects models.

To address our first research question, we explore variability in parent-child talk produced in the post-test phase by condition. To examine our second research question, we explore potential relations between parental talk and child-level talk.

### Quantity of Talk by Condition

Parents in the *scientific statements* condition (*M* = 66.41, *SD* = 37.00) used a marginally larger number of utterances than parents in the *scientific inquiry* condition (*M* = 49.12, *SD* = 26.26), *t*(45) = 1.86, *p* = 0.07. Children’s total utterances were not significantly different between the *scientific statements* condition (*M* = 20.73, *SD* = 20.99) and the *scientific inquiry* condition (*M* = 19.76, *SD* = 19.28), *t*(45) = 0.17, *p* = 0.87. Similarly, the amount of overall time dyads spent on task did not differ between the *scientific inquiry* condition (*M* = 4.65 min, *SD* = 2.16 min) and the *scientific statements* condition (*M* = 5.49 min, *SD* = 3.36 min), *t*(45) = −1.04, *p* = 0.31. To investigate the possibility that the quantity of parent and child talk differed as a function of child age, two linear regressions were run, predicting the total number of child and parent utterances from child age as a continuous variable. The parent model showed a significant effect of child age (β = −12.48, *SE* = 5.38, *p* = 0.03) on total parent utterances, *F*(1, 45) = 5.38, *p* = 0.03, *R*^2^ = 0.11. This indicates that parental talk was negatively associated with child age. The child model did not show a significant effect of child age (*B* = −0.18, *SE* = 3.48, *p* = 0.96) on total child utterances, *F*(1, 45) = 0.003, *p* = 0.96, *R*^2^< 0.001.

### Parent Talk by Delivery and Content

To explore the impact of training condition on parents’ language, we conducted planned binary-logistic mixed-effects models on the likelihood that a given utterance is a question or statement, with Condition (scientific inquiry, scientific statements; statements was the reference category) and Content (causal, fact-based, procedural; procedural was the reference category for *Model 1* and fact-based was the reference category for *Model 2*) as predictors, and dyad as a random factor to account for individual variance. See Table 2 (Model 1) for a summary of parent results and Table 3 (Model 1) for a summary of child results.

**TABLE 2:** Mixed effects binary-logistic regressions on parents’ likelihood to ask a question.

				**95% CI for *OR***	
**Variable**	**β (*SE*)**	***Z***	**Odds ratio**	**Lower**	**Upper**
**Model 1**					
Intercept	0.63 (0.16)***	−3.907	0.54	0.39	0.73
Condition (scientific statements as reference)	0.52 (0.22)*	2.35	1.68	1.09	2.63
Causal Content (procedural as reference)	0.50 (0.20)*	2.50	1.65	1.12	2.45
Fact-based content (procedural as reference)	0.23 (0.16)	1.48	1.26	0.93	1.72
Condition × Causal content	0.90 (0.30)**	3.03	2.46	1.38	4.41
Condition × Fact-based content	−0.23 (0.24)	−0.97	0.80	0.50	1.26
−2LL	−1121.7				
AIC	2257.50				
**Model 2**					
Intercept	−0.39 (0.16)*	−2.39	0.68	0.49	0.93
Condition (scientific statements as reference)	0.29 (0.24)	1.22	1.34	0.84	2.16
Causal content (fact-based as reference)	0.27 (0.20)	1.33	1.31	0.88	1.95
Procedural content (fact-based as reference)	0.23 (0.24)	−1.48	0.79	0.58	1.08
Condition × Causal content	1.13 (0.31)***	3.64	3.09	1.69	5.70
Condition × Procedural content	0.23 (0.24)	0.97	1.26	0.79	2.00
−2LL	−1121.70				
AIC	2257.50				

*p < 0.05, **p < 0.01, ***p < 0.001. Statements were used as a reference group. CI = 95% confidence interval; OR = odds ratio.

**TABLE 3:** Mixed effects binary logistic regressions on children’s likelihood to ask a question.

				**95% CI for *OR***	
**Variable**	**β (*SE*)**	***Z***	**Odds ratio**	**Lower**	**Upper**
**Model 1**					
Intercept	−2.16 (0.37)***	−5.85	0.12	0.05	0.22
Condition (scientific statements as reference)	−0.31 (0.57)	−0.54	0.74	0.22	2.15
Causal Content (procedural as reference)	1.83 (0.47)***	3.93	6.26	2.55	16.25
Fact-Based Content (procedural as reference)	−0.34 (0.49)	−0.71	0.71	0.26	1.82
Condition × Causal Content	−1.31 (0.74)^t^	−1.78	0.27	0.06	1.17
Condition × Fact-Based Content	−0.28 (0.74)	−0.38	0.76	0.18	3.29
−2LL	−180.40				
AIC	374.80				
**Model 2**					
Intercept	−2.50 (0.42)***	−5.976	0.08	0.03	0.17
Condition (scientific statements as reference)	−0.59 (0.59)	−1.00	0.56	0.17	1.77
Causal Content (fact-based as reference)	2.18 (0.53)***	4.09	8.83	3.18	26.36
Procedural Content (fact-based as reference)	0.28 (0.74)	0.71	1.41	0.55	3.80
Condition × Causal Content	−1.03 (0.77)	−1.34	0.36	0.08	1.61
Condition × Procedural Content	0.23 (0.24)	0.70	1.32	0.30	5.56
−2LL	−1121.70				
AIC	2257.50				

^*t*^p = 0.08, ***p < 0.001. Statements were used as a reference group. CI, 95% confidence interval; OR, odds ratio.

#### Model 1: Effects of Condition by Content

A binary-logistic mixed-effect model on likelihood that a parent’s utterance is a question found a significant main effect of Condition (β = 0.52, *SE* = 0.22, *p* = 0.02, *OR* = 1.68, 95% CI [1.09, 2.63]), a significant main effect of Causal Content (*B* = 0.50, *SE* = 0.20, *p* = 0.01, *OR* = 1.65, 95% CI [1.12, 2.45]), and a significant interaction between Condition and Causal Content (*B* = 0.90, *SE* = 0.30, *p* < 0.01, *OR* = 2.46, 95% CI [1.38, 4.41]). No other main effects or interactions were found. Utterances were 1.68 times more likely to be a question if the parent was trained in the *scientific inquiry* condition, 1.65 times more likely to be a question if the parent presented causal content in general, and 2.46 times more likely to be a question if the parent was in the *scientific inquiry* condition and was presenting causal content. See Table 2 (top panel) for a summary of this model. See Table 4 for model-estimated mean likelihoods from *Model 1*.

**TABLE 4:** Estimated likelihood that utterances are a question by condition and delivery.

	**Parents**		**Children**	
	**Scientific inquiry**	**Scientific statements**	**Scientific inquiry**	**Scientific statements**
Causal	0.79	0.47	0.13	0.42
Fact-based	0.48	0.40	0.04	0.08
Procedural	0.47	0.35	0.08	0.10

*Model-estimated mean likelihoods that an utterance of a specific content type is a question (as opposed to a statement) from parent Model 1 (Table 2), a binary-logistic mixed-effect model on likelihood that a parent’s utterance is a question and child Model 1 (Table 3), a binary-logistic mixed-effect model on likelihood that a child’s utterance is a question. For instance, in the scientific inquiry condition, the model estimates that there is a 79% likelihood of a causal utterance being posed as a question (as opposed to a statement).*

#### Model 2

To confirm the results of *Model 1*, the reference category for Content was changed to fact-based. This model found a significant interaction between Condition and Causal Content, *B* = 1.13, *SE* = 0.31, *p* < 0.001, *OR* = 3.09, 95% CI [1.69, 5.70]. Parents in the *scientific inquiry* condition were 3.09 times more likely than parents in the *scientific statements* condition to present causal content as questions. No other main effects or interactions were found. Table 2 (bottom panel) includes all model parameters.

To confirm that the conclusions of our mixed-model logistic regressions were found across individual dyads, we explored the number of dyads in the *scientific inquiry* condition who displayed more questions than statements when presenting causal content. Twenty-one out of 25 (84%) parents in this condition displayed this pattern. By contrast, only 7 out of 22 (31.81%) parents in the *scientific statements* condition displayed this pattern.

Taken together, both the binary-logistic mixed-models and individual inspection at the dyad level provide similar results. Parents’ utterances overall are more likely to be questions when the parent is both in the *scientific inquiry* condition and presenting causal content and a majority of dyads in the *scientific inquiry* condition displayed this pattern.

### Child Talk by Delivery and Content

To explore the impact of training condition on children’s language, we conducted planned binary-logistic mixed-effects models on the likelihood that a given utterance is a question or statement, with Condition (scientific inquiry, scientific statements; statements was the reference category) and Content (causal, fact-based, procedural; procedural was the reference category for *Model 1* and fact-based was the reference category for *Model 2*) as predictors, and dyad as a random factor to account for individual variance.

#### Model 1: Effects of Condition by Content

A binary-logistic mixed-effect model on likelihood that a child’s utterance is a question found a significant main effect of Causal Content, *B* = 1.83, *SE* = 0.47, *p* < 0.0001, *OR* = 6.26, 95% CI (2.55, 16.25), and a marginally significant interaction between Condition and Causal Content, β = −1.31, *SE* = 0.74, *p* = 0.08, *OR* = 0.27, 95% CI [0.06, 1.17]. No other main effects or interactions were found. Compared to procedural talk, children’s utterances were 6.26 times more likely to be a question when discussing Causal Content. See Table 3 (top panel) for a summary of this model. See Table 4 for model-estimated mean likelihoods from *Model 1*.

#### Model 2

To confirm the results of *Model 1*, the reference category for Content was changed to be fact-based. This model found a significant main effect of Causal Content, *B* = 2.18, *SE* = 0.53, *p* < 0.0001, *OR* = 8.83, 95% CI [3.18, 26.36]. Compared to fact-based talk, children’s utterances were 8.83 times more likely to be a question when discussing Causal Content. No other main effects or interactions were found. Table 3 (bottom panel) includes all model parameters.

### Child Responses to Parental Causal Utterances

Finally, we explored potential condition-level differences on the relation between the type of parental talk and children’s subsequent responses. Recall that parents produced 74% of the overall talk in the post-training phase. We focused specifically on causal talk, as this was the type of talk content that was not only (1) targeted by both conditions but also (2) significantly differed between conditions according to parental delivery. Parental causal utterances were individually coded according to two criteria: (1) whether or not a child provided a “response” (i.e., a child utterance immediately following a parent utterance) and (2) if there was a child response, whether the content of it was scientific in nature (fact-based or causal).

To explore potential condition-level differences, a mixed-model binary logistic regression was run on the likelihood of child responses to parental causal talk with Condition (*scientific inquiry, scientific statements*) as a fixed factor and dyad as a random factor. These results yielded a marginally significant main effect of Condition, *B* = 0.84, *SE* = 0.47, *p* = 0.07, *OR* = 2.32, 95% CI [0.90, 6.42]. No other main effects or interactions were found. Children in the *scientific inquiry* condition were 2.32 times more likely to respond to a parental causal utterance than children in the *scientific statements* condition. Table 5 includes all model parameters.

**TABLE 5:** Mixed effects binary logistic regressions on children’s likelihood to respond to parental causal talk.

				**95% CI for *OR***	
**Variable**	**β (*SE*)**	***Z***	**Odds ratio**	**Lower**	**Upper**
Intercept	−0.22 (0.35)***	−0.65	0.80	0.37	1.59
Condition (scientific statements as reference)	0.84 (0.47)^t^	1.80	2.32	0.90	6.42
−2LL	−166.90				
AIC	339.90				

^*t*^p = 0.08, ***p < 0.001. No response was used as a reference group. CI, 95% confidence interval; OR, odds ratio.

To confirm that this pattern held at the level of the dyad, we explored the number of children in each condition who were more likely than not to respond to parents’ causal talk (i.e., responded to a parental causal utterance more than 50% of the time). In the *scientific inquiry* condition, 17 out of 25 children in the *scientific inquiry* condition (68.0%) were more likely than not to respond to a parental causal utterance, as compared to 9 out of 21 children (40.9%) in the *scientific statements* condition.

We also explored whether there were Condition differences in the *content* of children’s responses to parental causal utterances. Recall that children’s responses were coded as “scientific” (causal or fact-based content) or procedural. An additional mixed-model binary logistic regression was run with Condition as a fixed factor and dyad as a random factor. A significant main effect of Condition was found, β = 1.83, *p* = 0.0005. No other main effects or interactions were found. When responding to parental causal utterances, children in the *scientific inquiry* condition were 6.22 times more likely to provide a scientific response (fact-based or causal) than children in the *scientific statements* condition. Table 6 includes all model parameters.

**TABLE 6:** Mixed effects binary logistic regressions on children’s likelihood to respond with scientific talk.

				**95% CI for *OR***	
**Variable**	**β (*SE*)**	***Z***	**Odds ratio**	**Lower**	**Upper**
Intercept	−1.53 (0.42)***	−3.61	0.22	0.08	0.46
Condition (scientific statements as reference)	1.83 (0.53)***	3.47	6.22	2.31	20.80
−2LL	−156.40				
AIC	318.80				

***p < 0.001. Procedural content was used as a reference group. Scientific talk was composed of causal or fact-based content. CI, 95% confidence interval; OR, odds ratio.

To confirm that this pattern held at the level of the dyad, we explored the number of children in each condition who were more likely to respond with a scientific utterance (i.e., provided a scientific response more than 50% of the time) to parent’s causal talk. In the *scientific inquiry* condition, 13 out of 25 children in the *scientific inquiry* condition (52%) were more likely than not to provide a scientific response to a parental causal utterance, as compared to 2 out of 21 children (9.52%) in the *scientific statements* condition.

## Discussion

In this study, we investigated impacts of a brief pedagogical intervention on both parents’ and children’s conversation when interacting with scientific activities in a museum context. We also examined how parents’ delivery of the causal content targeted by this pedagogical training influenced children’s responsiveness to the parent while discussing scientific concepts. Below, we review these findings before discussing implications for parent-child interactions during informal science learning settings, as well as avenues for future research.

Immediately following the short 5 min training, parents’ delivery of utterances differed between conditions. However, the content of those utterances did not. When interacting with the gears with their children, parents in both conditions engaged in explanatory talk that included causal language, yet the process by which that language was delivered varied by condition. Whereas parents in the *scientific inquiry* condition asked more causal questions, parents in the *scientific statements* condition used more causal statements. Importantly, results from a series of mixed-effects regression models indicated that this condition difference was not found when parents engaged in fact-based or procedural talk. These results indicate that parents’ adherence to the modeled pedagogical approaches was specific to the content *targeted* by those approaches – causal scientific concepts, discussing or prompting consideration of the relation between scientific observations, and features of scientific processes.

In addition to impacting parent-level talk, the training session also had effects at the child level. Specifically, children were more likely to respond to parental causal talk in the scientific inquiry condition than in the scientific statements condition. Such responses indicate that parent-child conversation in this condition was more elaborative and included more turns per individual topic. Moreover, when they did respond to their parents’ causal talk, children were significantly more likely to produce on-topic scientific responses rather than procedural responses. As a result, parents and children are more likely to generate joint opportunities to learn about specific scientific content in the scientific inquiry condition. This finding complements and extends the research by Gutwill and Allen (2010) and Vandermaas-Peeler et al. (2016) by highlighting how inquiry processes can prompt extended conversations about science between parents and children, illustrating the important role that social interaction plays for children’s scientific learning. Indeed, recent research has highlighted the importance of extended conversational turns with parents for children’s language outcomes (Romeo et al., 2018). Additionally, this study builds on previous work finding that children are more likely to respond to *Wh-* questions, which include causal questions, as compared to other types of questions (Rowe et al., 2017). The current study highlights how conversational turns might also be important for strengthening children’s domain knowledge. However, it is important to note that the low child response rate overall prevented further exploration via mediation analyses into the extent to which aspects of both the delivery and content most affected child response rates. Future research should explore this question further.

Parents’ willingness to adopt both the delivery and content targeted by the short modeled training is consistent with previous training studies focusing on modifying specific targeted language (e.g., *Wh*- questions: Benjamin et al., 2010; Haden et al., 2014) or modifying inquiry processes at the family level (Gutwill and Allen, 2010). As we indicate above, our findings replicate and extend this work to highlight that such guidance has effects at both the parent level and the child level and not only for specific content but also for the process through which that content is delivered. Importantly, findings from the current study were found in a sample of parent-child dyads with children who were considerably younger than those included in previous informal science learning interventions (e.g., Gutwill and Allen, 2010; Vandermaas-Peeler et al., 2016, 2017, 2018). Thus, inquiry-based approaches appear to be effective during the developmental period when parents are children’s primary teachers – prior to their child’s extensive experiences with formal education.

The impact of the short training session on parent-level and child-level talk is all the more striking when considering that the training generalized across tasks (from an electrical circuit to gears) and was found in spontaneous, self-generated speech. Recall that any utterances that specifically referenced the post-training supplemental information sheet were not included in analyses. These data are consistent with some recent research indicating impacts of parental causal talk on children’s ability to generalize causal mechanisms when interacting with novel physical stimuli (Kurkul et al., under review; Leech et al., 2019). Future research should explore how such an intervention not only impacts the explanatory talk by parents and children but also impacts child-level learning outcomes.

This study has several notable limitations. The first was that the effectiveness of the short training on parent-child conversation was explored immediately following the training session. Some research has indicated impacts of interventions up to 1 month later (Gutwill and Allen, 2010; Haden, 2010; Haden et al., 2014) and so it is plausible that such short interventions can have longer-lasting effects. Future research should explore the extent to which dyads continue to show training effectiveness on parent-child conversation with a time delay, or across other contexts beyond the museum. Second, as we tested our intervention in the context of a gears activity, it is unknown whether our scientific inquiry approach would be more or less successful across other science activities. Third, as this study aimed to compare the efficacy of the scientific inquiry and scientific statements approaches in eliciting child engagement in scientific conversations, it did not utilize a pre-test–post-test design or a direct measure of child learning outcomes. As such, our findings do not reveal whether parents’ natural interaction styles were modified by the intervention or whether children learned specific scientific concepts or facts.

Finally, because data were collected from a sample of parent-child dyads in a science museum, most participants likely had an interest in science and the majority were likely to be of middle-to-high socioeconomic status. Future research should explore the effectiveness of these training interventions on a more diverse sample of families. Dyadic inquiry processes and conversations vary considerably based on family background (Tizard and Hughes, 1984; Bang and Medin, 2010; Coppens et al., 2014; Rogoff, 2014; Solis and Callanan, 2016; Kurkul and Corriveau, 2018; Gauvain and Munroe, 2020). Therefore, the inquiry processes modeled here may be less familiar to some families, making the practices more or less difficult to integrate into typical conversational patterns. Indeed, family inquiry can take many forms, and may not always be displayed in the manner we have defined inquiry in this study. In some communities, a different type of inquiry might be displayed as children “listening in” or engaging in non-verbal observation of others (e.g., Rogoff, 2003). In these or other communities, question-asking to adults is not common or expected (Gauvain, 2001; Johnston and Wong, 2002; Rogoff, 2003; Gauvain and Munroe, 2020). Future research should explore how best to adapt this training session to the discourse patterns of diverse family backgrounds to promote engaging scientific conversations between parents and children.

Taken together, this study provides evidence that a brief training in scientific inquiry helps parents and children talk about higher-order scientific causal content in a collaborative manner in the context of a science museum. Training parents to talk with their children regarding the causal relations underpinning scientific concepts has the potential to have far-reaching impacts on children’s interest in and learning of scientific concepts prior to their introduction to formal schooling. Future research should further explore the full implications of scientific inquiry training and other similar methodologies on parent-child dyadic talk in informal learning settings.

## Data Availability Statement

A de-identified version of the dataset used for multi-level analyses is available on request to the corresponding author.

## Ethics Statement

The studies involving human participants were reviewed and approved by the Boston University Institutional Review Board, Charles River Campus. Written informed consent to participate in this study was provided by the participants’ legal guardian.

## Author Contributions

IC-C wrote the first draft of the manuscript and collected the data. KL and KC provided substantial feedback. KC and IC-C designed the study. IC-C, KL, and KC designed the coding and transcription schemes, and conducted data analysis. All authors contributed to the article and approved the submitted version.

## Conflict of Interest

The authors declare that the research was conducted in the absence of any commercial or financial relationships that could be construed as a potential conflict of interest.
